# The role of smoking cessation programs in lowering blood pressure: A retrospective cohort study

**DOI:** 10.18332/tid/142664

**Published:** 2021-10-22

**Authors:** Szu-Ying Tsai, Wei-Hsin Huang, Hsin-Lung Chan, Lee-Ching Hwang

**Affiliations:** 1 Department of Family Medicine, Mackay Memorial Hospital, Taipei City, Taiwan; 2 Department of Family Medicine, Taipei City Hospital, Zhongxing Branch, Taipei City, Taiwan; 3 Department of Medicine, Mackay Medical College, New Taipei City, Taiwan

**Keywords:** hypertensive, cigarette, diastolic blood pressure, smoking cessation

## Abstract

**INTRODUCTION:**

Cigarette smoking affects blood pressure and is a major risk factor for cardiovascular diseases. The role of smoking cessation programs with respect to blood pressure remains inconclusive. Thus, this study aimed to investigate the effects of a smoking cessation program on blood pressure.

**METHODS:**

Participants who attended the smoking cessation program in an outpatient clinic of a tertiary medical center in Taiwan from 2017 to 2018 were enrolled in this retrospective cohort study. Their smoking cessation status was traced via phone calls during the third month, and the researchers collected participant characteristics and blood pressure before and after the program. Differences in the participants’ blood pressure, based on those with and those without hypertension, were compared using analysis of covariance. Univariable logistic regression models were used to determine factors associated with success in smoking cessation. In total, there were 721 participants. The participants had a mean age of 55.8±11.4 years and 68.1% of the participants were hypertensive.

**RESULTS:**

During the program, the overall systolic blood pressure decreased by 4.0±17.9 mmHg and diastolic blood pressure decreased by 2.5±12.0 mmHg, from the baseline. Hypertensive participants showed a more prominent blood pressure lowering effect compared to non-hypertensive participants in terms of the subtraction difference of systolic blood pressure (-5.0±19.0 vs -1.9±15.2 mmHg, p=0.018) and diastolic blood pressure (-3.1±12.9 vs -1.1±9.6 mmHg, p=0.016). After multivariate control, the results showed that the adjusted subtraction difference of diastolic blood pressure was still more significant in the hypertensive group than in the non-hypertensive group.

**CONCLUSIONS:**

The smoking cessation program significantly reduced both systolic blood pressure and diastolic blood pressure in the entire cohort. The results were more significant in the hypertensive group compared to the non-hypertensive group.

## INTRODUCTION

Hypertension is a global health issue^1^. High blood pressure can lead to organ damage^2^, cardiovascular diseases (CVDs)^3^, and even premature death^4^. It affects more than 1 billion people worldwide^5^; indeed, it has become a global epidemic that is considered a major public health issue^6^.

Cigarette smoking influences some cardiovascular parameters, including blood pressure^7-9^. However, research results on the relationship between smoking cessation and blood pressure vary. Some researchers found a paradoxical association between cigarette smoking and blood pressure, with current smokers showing lower blood pressure than ex-smokers^7,10^ or non-smokers^10,11^. In one study, smokers had higher daytime ambulatory systolic blood pressure than non-smokers^12^. However, in another study, blood pressure did not differ among current smokers, past smokers, and non-smokers^13^. Smoking increases the risk of coronary, cerebrovascular, and renal diseases in hypertensive patients and is considered a major modifiable risk factor for hypertension^14^.

According to World Health Organization (WHO) estimates, tobacco kills more than 7 million people each year. The WHO has been dedicated to tobacco cessation and the WHO Framework Convention on Tobacco Control addressed a policy of accessible and free or affordable medication provided by national policy or health insurance^15^. To alleviate the health hazard of tobacco use, it is necessary that regional healthcare systems provide tobacco abstinence programs.

In Taiwan, the prevalence of smoking among adults was approximately 22% in 2008^16^, and 14.5% in 2017^17^. However, despite the steady observed decline, major economic losses related to tobacco use remain high at approximately 50 billion New Taiwan dollars annually^18^. Historically, Taiwan established national health insurance in 1995, and 99.6% citizens have joined to date^19^. Because of the high prevalence of tobacco use and its associated health threats, the Ministry of Health and Welfare initiated a smoking cessation program in 2002 paid by the national health insurance. The outpatient program was designed to encourage doctors to integrate smoking cessation counseling in routine outpatient visits^16^. Doctors who attended the 8-hour workshop and passed the exam became qualified to hold smoking cessation outpatient service programs in clinical practice. Smokers who visited the clinics received counseling from a doctor in the 5As (ask, advise, assess, assist, arrange), 8 weeks of pharmacotherapy (including varenicline, nicotine replacement therapy, or bupropion) for a maximum of two courses per year, and health education including tobacco abstinence skills and feedback during the course from trained and certificated nurses or counselors. The counselors conducted a telephone follow-up after 3 months, and participants underwent a thorough interview to provide updates on their cessation status and dynamics as well as to give feedback 6 months after the program ended. The participants returned to clinics every 2 to 4 weeks for pharmacological therapy, which was affordable at a maximum of 200 New Taiwanese dollars (about 6.85 US$) per visit. The smoking cessation program was established properly, and studies showed that rates of receiving quitting advice increased among all smokers^16^. The number of participants in the smoking cessation program increased from 13142 in 2002 to over 0.7 million in 2018^16,17^.

The relationship between smoking cessation and blood pressure is controversial^7-9,11,12^. Epidemiological research has reported different results in this regard^20^. Some found a positive association between cigarette smoking and blood pressure, whereas others found a possible negative correlation. However, findings are inconsistent, in part, due to the lack of experimental data. Therefore, we sought to clarify the relationship between the two in this cohort study and resolve the discrepancies in the previous findings. At the same time, we sought to elucidate whether and how a well-established smoking cessation program could contribute to healthier outcomes. Because there have been so few reports on the health outcomes of participants^16,21,22^, we attempted to measure the effects of attending a health promotion program, specifically a smoking cessation program, on blood pressure.

## METHODS

### Study population

This was a retrospective cohort study. We searched the database at our institution for patients who had attended the smoking cessation program in the outpatient clinics at the Mackay Memorial Hospital, Taipei, Taiwan, from January 2017 to December 2018. For the study, we selected 721 participants aged 18–74 years, with complete data and physical parameters considered. We divided the participants into two groups: the non-hypertensive group and the hypertensive group. The hypertensive group consisted of those who had been diagnosed with hypertension or with the disease code I10 in The International Statistical Classification of Diseases and Related Health Problems 10th Revision (ICD-10) according to their medical records at Mackay Memorial Hospital and patients who were currently using antihypertensive medication.

### Study protocol and data collection

We retrieved the participants’ basic characteristics such as gender, age, pack-years (packs per day × years), the number of daily cigarettes, and the Fagerström test of nicotine dependence (FTND) score^23^, using a questionnaire administered by trained nurses, during the participants’ first smoking cessation outpatient clinic visit. FTND scores range from 0 to 10 and reflect nicotine dependence, with higher scores representing greater dependence. Smokers with FTND scores ≥7 will possibly have strong withdrawal symptoms and relapse early^23,24^.

Trained nurses also measured the participants’ baseline body weight (kg) and exhaled carbon monoxide (ppm) concentrations. Doctors measured the patients’ rest blood pressure using a sphygmomanometer after a 10-minute rest, and participants were categorized as having cardiovascular diseases (CVDs) if the team noted any of the following conditions or diagnoses in the data sets or in the patients’ medical records: ST-segment elevation or non-ST-segment elevation myocardial infarction, CVDs with stent, any stroke, angina, ischemic heart disease, or peripheral arterial diseases. Participants were categorized as having diabetes if we found a diagnosis of diabetes or a record of any antidiabetic medication in the data sets or in their medical records.

The smoking cessation program is well-established in the clinic, and every patient who joins the clinic receives information on the program, a clear concept of lifestyle modification, and the healthy lifestyle they need to adopt. In the hospital, we conducted the smoking cessation program in the outpatient clinics of family physicians and internal medicine physicians. Participants were to provide an accurate abstinence date at their first clinical visit. Doctors prescribed varenicline or nicotine replacement therapy after evaluation, despite contraindications such as allergies. The patients were also asked to throw away all their cigarettes and lighters. Certificated counselors or nurses arranged to interview the participants in person, provide abstinence skills and advise them to declare on social media and on their personal web pages that they were quitting smoking and to tell their families and friends. The counselors also asked the participants to avoid places with a chance of tobacco exposure, and advised them that if they thought about smoking, they could distract themselves by drinking water, taking a deep breath, or doing some exercise. The counselors also encouraged a regular daily routine and sleep, and the patients were scheduled to return to the clinic every 2 to 4 weeks during medication use.

We obtained data on the participants’ final blood pressure, exhaled concentration of carbon monoxide (ppm), final daily cigarettes and body weight within 3 months after attending the program. The counselors also followed up with the patients via telephone to track their smoking status during the third month. During thorough interviews, the counselors asked the patients to confirm their smoking status, specifically whether they had smoked during the previous 2 weeks. If the patient answered ‘yes’ then the patient was considered to have failed the smoking cessation program, whereas a response of ‘no’ was confirmed as success.

### Data analysis and outcome measurement

We calculated mean arterial pressure using systolic and diastolic blood pressure. We also calculated the differences between the patients’ baseline and follow-up at 3 months values for body weight (including the percent difference), daily number of cigarettes, and both the systolic and diastolic blood pressure (including percent difference).

We conducted a series of univariate analyses of the quantitative data using IBM SPSS version 20, and the two-tailed test significance level was set at p<0.05. We used independent t-test to calculate the continuous variables, such as the basic demographic data, with the variables presented as either mean ± SD or percent difference. We used the chi-squared test for the categorical variables, which are presented as frequencies and percentages. We used analysis of covariance (ANCOVA) to calculate the means and percent differences in the participants’ blood pressure, both with and without hypertension, adjusting for age, gender, diabetes, CVD, FTND score, pack-years, successful smoking cessation during the 3 months after the program, varenicline use and antihypertensive medication use during the 3-month follow-up period, body weight percent difference, and total number of outpatient clinic visits. We reviewed the data sets for the antihypertensive medications, and the patient was considered as using antihypertensive medication if we found a record of any angiotensinconverting enzyme inhibitors, angiotensin receptor blockers, direct renin inhibitors, beta-blockers, calcium-channel blockers, alpha-blockers, diuretics, or direct-acting vessel dilators for at least 28 consecutive days. Finally, we used univariable logistic regression models to determine whether blood pressure, reduced blood pressure, and the difference between the two, were associated with success in smoking cessation.

## RESULTS

There were 721 participants in this study. Their basic characteristics are presented in Table 1. The participants were aged 55.8±11.4 years on average, 627 (87.0%) were men and 491 (68.1%) were hypertensive.

**Table 1:** Demographic data of study subjects, Taipei, Taiwan, 2017–2018 (N=721)

*Characteristics*	*Total Mean±SD*	*HTN group (n=491) Mean±SD*	*Non-HTN group (n=230) Mean±SD*	p*
Age (years)	55.8±11.4	57.6±10.5	52.1±12.2	<0.001
Male, n (%)	627 (87.0)	451 (91.9)	176 (76.5)	<0.001
Baseline body weight (kg)	70.8±18.9	73.1±18.2	65.9±19.2	<0.001
Pack-years	32.9±12.4	34.7±11.8	29.0±12.9	<0.001
Daily cigarette count (sticks)	21.3±40.9	22.3±48.9	19.1±11.8	0.337
Baseline SBP (mmHg)	132.9±18.7	135.9±19.1	126.5±16.0	<0.001
Baseline DBP (mmHg)	79.5±13.0	81.2±13.6	75.9±10.6	<0.001
Baseline MAP (mmHg)	97.3±14.0	99.4±14.6	92.7±11.4	<0.001
DM, n (%)	232 (32.2)	179 (36.5)	53 (23.0)	<0.001
CVD, n (%)	235 (32.6)	198 (40.3)	37 (16.1)	<0.001
Baseline exhaled carbon monoxide (ppm)	12.1±10.8	12.4±10.4	11.7±11.8	0.508
FTND score	6.2±2.4	6.0±2.4	6.5±2.3	0.018

* Using chi-squared and t-test. Statistical significance was defined as p<0.05. HTN: hypertension. SBP: systolic blood pressure. DBP: diastolic blood pressure. MAP: mean arterial blood pressure. DM: diabetes mellitus. CVD: cardiovascular disease. FTND: Fagerström test of nicotine dependence.

We further analyzed the hypertensive and nonhypertensive groups separately, and their average age was 57.6±10.5 years and 52.1±12.2 years, respectively (p<0.001). There were 451 (91.9%) men in the hypertensive group and 176 (76.5%) in the nonhypertensive group (p<0.001). The body weight was higher in the hypertensive group than in the nonhypertensive group (73.1±18.2 vs 65.9±19.2 kg, p<0.001). The average pack-years were 34.7±11.8 and 29.0±12.9 in the hypertensive and non-hypertensive groups, respectively (p<0.001). Participants in the hypertensive group had a lower average FTND score (6.0±2.4) than the non-hypertensive group (6.5±2.3) (p=0.018), and baseline systolic and diastolic blood pressure values were higher in the hypertensive group (135.9±19.1 mmHg and 81.2±13.6 mmHg) than in the non-hypertensive group (126.5±16.0 mmHg and 75.9±10.6 mmHg) (p<0.001). Participants with hypertension also showed a higher incidence of diabetes (p<0.001) and CVD (p<0.001) (Table 1).

By the third month, 228 participants (31.6%) had successfully quit smoking. As shown in Table 2, there was no significant difference between the hypertensive and the non-hypertensive groups in the third month smoking cessation rate (31.8% for hypertensive and 31.3% for non-hypertensive).

**Table 2:** Comparisons of characteristics between HTN group and Non-HTN group after attending smoking cessation, Taipei, Taiwan, 2017–2018 (N=721)

*Variable*	*Total Mean±SD*	*HTN group (n=491) Mean±SD*	*Non-HTN group (n=230) Mean±SD*	p*
Final body weight (kg)	70.7±19.3	73.4±18.0	65.0±20.6	<0.001
Body weight subtraction difference (kg)^a^	-0.1±7.2	0.3±6.1	-0.9±9.3	0.043
Body weight percent difference (%)^b^	-0.4±7.8	0±5.0	-1.2±11.7	0.157
Outpatient clinics visits	2.6±1.5	2.7±1.5	2.3±1.3	0.005
No tobacco cessation medication, n (%)	92 (12.8)	54 (11.0)	38 (16.5)	<0.001
Nicotine replacement therapy, n (%)	79 (11.0)	43 (8.8)	36 (15.7)	<0.001
Varenicline use, n (%)	550 (76.3)	394 (80.2)	156 (67.8)	<0.001
Tobacco cessation drug duration (weeks)	4±2.9	4.1±2.9	3.8±2.9	0.331
Daily cigarette minus count (sticks)	9.7±41.2	10.6±49.3	7.8±11.6	0.392
Success in daily cigarette minus, n (%)	284 (39.4)	197 (40.2)	87 (37.8)	0.543
Success in quitting smoking by 3rd month, n (%)	228 (31.6)	156 (31.8)	72 (31.3)	0.900
Final exhaled carbon monoxide (ppm)	13.4±10.7	13.3±9.3	13.7±13.0	0.816
Subtraction difference of exhaled carbon monoxide (ppm)^c^	-3.9±10.2	-4.0±8.6	-3.7±12.7	0.873
Use of anti-hypertensive medication, n (%)	423 (58.7)	420 (85.5)	3(1.3)	NV
Final SBP (mmHg)	128.9±15.5	130.9±14.8	124.6±16.1	<0.001
Final DBP (mmHg)	77.0±10.9	78.1±11.1	74.8±10.0	<0.001
SBP subtraction difference (mmHg)^d^	-4.0±17.9	-5.0±19.0	-1.9±15.2	0.018
SBP percent difference (%)^e^	-2.0±12.8	-2.5±13.0	-0.8±13.2	0.095
DBP subtraction difference (mmHg)^f^	-2.5±12.0	-3.1±12.9	-1.1±9.6	0.016
DBP percent difference (%)^g^	-1.8±14.6	-2.4±14.8	-0.4±13.9	0.076
Success in lowering SBP, n (%)	374 (51.9)	286 (54.6)	106 (46.1)	0.033
Success in lowering DBP, n (%)	353 (49.0)	252 (51.3)	100 (43.5)	0.049

* Using chi-squared and the t test. NV: No value because the target chi-squared was less than 5. Statistical significance was defined as p<0.05. HTN: hypertension. SBP: systolic blood pressure. DBP: diastolic blood pressure.

a Final body weight within 3 months minus the baseline body weight.

b Body weight subtraction difference divided by the baseline body weight.

c Final exhaled carbon monoxide within 3 months minus the baseline exhaled carbon monoxide.

d Final SBP within 3 months minus the baseline SBP.

e SBP subtraction difference divided by the baseline SBP.

f Final DBP within 3 months minus the baseline DBP.

g DBP subtraction difference divided by the baseline DBP.

After the program, 374 (51.9%) participants recorded lower systolic blood pressure and 353 (49.0%) recorded lower diastolic blood pressure. In terms of the differences, the subtraction difference for systolic blood pressure was as high as -4.0±17.9 mmHg, and the percent difference was -2.0±12.8%; for diastolic pressure, the subtraction difference was -2.5±12.0 mmHg on average and the percent difference was -1.8±14.6%. At the end of the program, 286 (54.6%) patients in the hypertensive group recorded lower systolic blood pressure and 252 (51.3%) recorded lower diastolic blood pressure compared with the non-hypertensive group that showed 106 (46.1%; p=0.033) participants with lower systolic blood pressure and 100 (43.5%; p=0.049) with lower diastolic blood pressure. The subtraction difference for systolic blood pressure was -5.0±19.0 mmHg in the hypertensive group and -1.9±15.2 mmHg in the non-hypertensive group (p=0.018), and the percent difference for systolic blood pressure was -2.5±13.0 % in the hypertensive group and -0.8±13.2% in the non-hypertensive group (p=0.095). The subtraction difference for diastolic blood pressure was -3.1±12.9 mmHg in the hypertensive group and -1.1±9.6 mmHg in the non-hypertensive group (p=0.016), and the percent difference for diastolic blood pressure was -2.4±14.8% in the hypertensive group and -0.4±13.9% in the non-hypertensive group (p=0.076) (Table 2).

After control for the covariates of sex, age, diabetes, CVD, FTND score, pack-years, successful smoking cessation during the third month, varenicline use, percent difference of body weight, total number of outpatient clinics visit, use of antihypertensive medication and the independent variable of hypertension, the results showed a non-significant difference in adjusted systolic blood pressure subtraction difference between the hypertensive (-5.4±1.0 mmHg) and the non-hypertensive (-1.2±1.9 mmHg) (p=0.094) groups after the smoking cessation program. The results also showed a non-significant difference between the hypertensive and nonhypertensive groups in the adjusted systolic blood pressure percent difference (hypertensive: -2.7±0.8%; non-hypertensive: -0.5±1.3%; p=0.214). The adjusted subtraction difference for diastolic blood pressure was significantly different between the hypertensive group (-3.6±0.7 mmHg) and the non-hypertensive group (-0.6±0.8 mmHg) (p=0.043). The adjusted percent difference for diastolic blood pressure was also non-significantly different between the hypertensive group (-2.8±0.9%) and the non-hypertensive group (0.1±1.5%) (p=0.151) (Table 3).

**Table 3:** The subtraction difference of SBP and DBP after analysis of covariance, Taipei, Taiwan, 2017–2018 (N=721)

*Variable*	*HTN group Mean±SD*	*Non-HTN group Mean±SD*	p*
The SBP subtraction difference (mmHg)^a^	−5.4±1.0	−1.2±1.9	0.094
The SBP percent difference (%)^b^	−2.7±0.8	−0.5±1.3	0.214
The DBP subtraction difference (mmHg)^c^	−3.6±0.7	−0.6±0.8	0.043*
The DBP percent difference (%)^d^	−2.8±0.9	0.1±1.5	0.151

* Using analysis of covariance. Statistical significance was defined as p<0.05. Covariates: sex, age, DM, CVD, FTND score, pack-year history, success smoking cessation during the 3rd month, varenicline use, body weight percent difference, total number of outpatient clinic visits, use of anti-hypertensive medication. HTN: hypertension. SBP: systolic blood pressure. DBP: diastolic blood pressure.

a Final SBP within 3 months minus the baseline SBP.

b SBP subtraction difference divided by the baseline SBP.

c Final DBP within 3 months minus the baseline DBP.

d DBP subtraction difference divided by the baseline DBP.

Whether and by how much systolic or diastolic blood pressure decreased were not related to the smoking cessation rate in the third month (Table 4). The factors associated with success in lowering blood pressure from the baseline during the smoking cessation program are shown in Supplementary file Table S1. We found no significant factors associated with lowered blood pressure across all subjects, although older patients were less likely to show lower blood pressure than their baseline values in the hypertensive group.

**Table 4:** The association between blood pressure change and success in smoking cessation, Taipei, Taiwan, 2017–2018 (N=721)

*Variable*	*Quitters Mean±SD*	*Non-quitters Mean±SD*	*OR (95% CI)*
Age (years)	56.3±10.9	55.6±11.6	1.00 (0.99–1.02)
Male (Ref.: Female), n (%)	203 (80.9)	424 (86)	1.32 (0.81–2.15)
The subtraction difference of exhale carbon monoxide (ppm)	-6.6±8.8	-2.1±10.7	0.95 (0.91–0.99)
The body weight subtraction difference (kg)^a^	-0.1±5.8	-0.1±7.8	1.00 (0.98–1.02)
The body weight percent difference (%)^b^	-0.1±6.8	-0.5±8.2	1.01 (0.98–1.03)
Baseline SBP (mmHg)	132.7±20.0	133.0±18.1	1.00 (0.99–1.00)
Baseline DBP (mmHg)	80.0±13.7	79.3±12.6	1.00 (1.00–1.00)
Final SBP (mmHg)	128.2±14.4	129.2±12.0	1.00 (1.00–1.00)
Final DBP (mmHg)	77.1±10.5	77.0±11.0	1.00 (1.00–1.00)
Success in lowering SBP, n (%)	115 (50.4)	259 (52.5)	0.90 (0.70–1.30)
Success in lowering DBP, n (%)	112 (49.1)	240 (48.7)	1.00 (0.70–1.40)
The SBP subtraction difference (mmHg)^c^	-4.6±20.4	-3.8±16.7	1.00 (1.00–1.00)
The SBP percent difference (%)^d^	-2.0±14.3	-2.0±12.0	1.00 (0.99–1.01)
The DBP subtraction difference (mmHg)^e^	-2.9±14.1	-2.3±10.8	1.00 (1.00–1.00)
The DBP percent difference (%)^f^	-1.9±15.8	-1.7±14.0	1.00 (0.99–1.01)

OR: odds ratio; odds ratio and 95% confidence interval (CI) after univariate logistic regression. HTN: hypertension. SBP: systolic blood pressure. DBP: diastolic blood pressure.

a Final body weight within 3 months minus the baseline body weight.

b Body weight subtraction difference divided by the baseline body weight.

c Final SBP within 3 months minus the baseline SBP.

d SBP subtraction difference divided by the baseline SBP.

e Final DBP within 3 months minus the baseline DBP.

f DBP subtraction difference divided by the baseline DBP.

## DISCUSSION

The results of this study revealed that during the smoking cessation program, both systolic and diastolic blood pressure improved significantly in the whole cohort. After we adjusted for the covariates, the diastolic blood pressure subtraction difference was greater in the hypertensive group than in the non-hypertensive group. As for the association between blood pressure subtraction difference during the program and the 3-month smoking cessation rate, we observed no significant changes. In fact, we could identify no specific factor associated with lowered blood pressure.

Meanwhile, education provided by doctors does play an important role in patients’ lifestyle changes. In this study, it is also possible that joining the program itself encouraged the participants to lead healthier lifestyles or to make an effort to quit smoking. We assumed that doctor education played an important role in changing the patients’ lifestyles and that it might have contributed to lowering blood pressure in the two groups.

Nicotine in cigarette smoke leads to an increase in a smoker’s blood pressure because it triggers the release of epinephrine and norepinephrine hormones^9^. A study involving diabetic patients revealed that both blood pressure and pulse rate decreased significantly after one month or later in the smoking cessation group^25^. In that retrospective cohort study involving 35 diabetic patients conducted at a smoking cessation clinic, systolic blood pressure significantly decreased by -7.0 mmHg during the first month (p=0.035) of the 12-month follow-up program^25^, but these results were only observed in the smoking cessation group, and their scale was small. Overall, previous research focused on the blood pressure changes between quitters and non-quitters and still yielded contradicting results^8,11^.

In the current study, we used ANCOVA to correct the group difference between hypertensive and non-hypertensive groups. Although the true mechanism remains unclear, we posit that arterial stiffness explains the blood pressure subtraction difference from baseline between the hypertensive and nonhypertensive groups^26^. Hypertension is a multifactorial disease, and one of the risk factors associated with it is arterial stiffness; in turn, blood pressure is a principal determinant of arterial stiffness^26-29^. In a previous study, duration of smoking cessation in ex-smokers had significant linear relationships with improved pulse wave velocity, augmentation index, and transit time, with arterial stiffness parameters returning to non-significant levels after a decade of smoking cessation^30^. In contrast, arterial stiffness might not directly affect blood pressure in non-hypertensive patients with only mild stiffness^26,31^. Therefore, we assumed in this study that the impact of the smoking cessation program in the non-hypertensive group would be less significant than that in the hypertensive group. However, further investigations are needed to explore this in depth.

The results of the current study also reveal that the blood pressure change observed during the smoking cessation program was not related to success in smoking cessation in either the hypertensive or the non-hypertensive group. Therefore, we went a step further to explore the physiological parameter changes during the smoking cessation program, and the results revealed significantly lower blood pressure, particularly in the hypertensive group. There are no previous studies that can help explain these results. We observed significantly lower blood pressure among all participants in the smoking cessation program in this study, but further research is needed to explore this.

### Strengths and limitations

One of the main strengths of this study is that the participants were recruited from a smoking cessation clinic that was visited by smokers; therefore, its findings can be applied to real cases. In terms of the study’s limitations, we collected the blood pressure data used herein randomly during the smoking cessation program, that is, not within a specific timeframe. It was also difficult to establish whether the participants had successfully quit smoking during the cessation program, and therefore, we could not draw any conclusions on whether a particular smoking status was associated with the lowered blood pressure we observed. Additionally, we recruited as participants the smokers who were attending a smoking cessation program, and the study findings would be strengthened with results from a control group who had not attended the program.

There are other limitations as well. First, we used a relatively small sample in this study, and a larger study cohort would yield more accurate results. Second, we retrieved the study participants’ blood pressure readings from their medical records, meaning the values had been measured at different times; therefore, the different readings could contain mistakes and bias. Third, we used a relatively short follow-up period after the smoking cessation program (only 3 months). A longer follow-up period would help future research to better evaluate the findings of such a study. Finally, we did not consider other possible confounding factors that could affect blood pressure, such as alcohol intake, exercise, diet, and salt intake; a similar study’s findings would be more robust if these factors were considered together.

## CONCLUSIONS

The findings of this study reveal that 51.9% of the patients who attended the smoking cessation program benefited in the form of lowered systolic and diastolic blood pressure. The effect was more significant among the participants in the hypertensive group than in the non-hypertensive group.

## Data Availability

The data supporting this research are available from the authors on reasonable request.
