# Supplementary material for: The role of smoking cessation programs in lowering blood pressure: A retrospective cohort study
Source: Tob Induc Dis. 2021 Oct 22;19:82. doi: 10.18332/tid/142664 (PMC8534426; doi:10.18332/tid/142664)
Supplement: Supplementary file 1 [file TID-19-82-s1.pdf]

Supplementary file

Table S1. The factors associated with success in blood pressure lowering from baseline during the smoking cessation program

	**Total Subjects**		**HTN Group**		**Non-HTN Group**	
	**N=721**		**N=491**		**N=230**	
	Success in lowering SBP	Success in lowering DBP	Success in lowering SBP	Success in lowering DBP	Success in lowering SBP	Success in lowering DBP
Gender (male, %)	0.79 (0.49,1.27)	0.73 (0.46,1.16)	1.38 (0.72,2.64)	1.67 (0.86,3.23)	1.12 (0.61,2.08)	1.32 (0.71,2.48)
Age (years)	1.00 (0.99,1.02)	1.00 (0.99,1.02)	0.98 (0.96,1.00)*	1.00 (0.98,1.01)	1.00 (0.98,1.03)	0.99 (0.97,1.01)
Varenicline use (n, %)	0.92 (0.62,1.36)	1.08 (0.74,1.58)	0.76 (0.48,1.19)	1.09 (0.69,1.70)	1.18 (0.68,2.06)	0.68 (0.39,1.19)
Outpatient clinics visit times (times)	0.98 (0.88,1.09)	0.98 (0.88,1.10)	0.90 (0.80,1.01)	0.98 (0.88,1.10)	0.98 (0.80,1.20)	1.06 (0.86,1.30)
The subtraction difference of exhaled carbon monoxide (ppm)†	1.00 (0.96,1.03)	0.98 (0.95,1.02)	1.05 (0.99,1.10)	1.02 (0.97,1.07)	0.99 (0.94,1.03)	1.01 (0.96,1.05)
Daily cigarette minus count (stick)	1.00 (0.99,1.01)	0.99 (0.98,1.01)	1.00 (1.00,1.01)	1.00 (1.00,1.01)	1.00 (0.98,1.03)	1.00 (0.98,1.02)
The body weight percent difference (%)	1.02 (0.99,1.04)	1.00 (0.98,1.02)	1.05 (0.96,1.15)	1.03 (0.97,1.09)	1.01 (0.98,1.03)	0.99 (0.97,1.02)
Use of anti-hypertensive medication (n, %)	1.19 (0.88,1.60)	1.22 (0.90,1.64)	0.75 (0.45,1.26)	0.79 (0.48,1.31)	2.37 (0.21,26.46)	--
Success in quitting smoke at 3rd month (n, %)	1.05 (0.74,1.50)	1.18 (0.84,1.65)	0.86 (0.58, 1.25)	1.04 (0.71, 1.52)	0.57 (0.23,1.38)	0.97 (0.56,1.70)

Odds ratio (OR) and 95% confidence intervals (CIs) are shown after univariate logistic regression. *Data are statistically significant ($p < 0.05$). †With missing data. - - Confidence interval too wide; Abbreviations: HTN, hypertension; SBP, systolic blood pressure; DBP, diastolic blood pressure; FTND, Fagerstrom Test of Nicotine Dependence; the body weight percent difference, the body weight subtraction difference divided by the baseline body weight; The subtraction difference of exhaled carbon monoxide, the final exhaled carbon monoxide record within 3 months minus the baseline exhaled carbon monoxide.

© 2021 Tsai S.Y. et al.
